# Greek Version of the Distress Thermometer for Parents of Children with Dysphagia: A Validation Study

**DOI:** 10.3390/jcm14124260

**Published:** 2025-06-15

**Authors:** Andri Papaleontiou, Vassiliki Siafaka, Louiza Voniati, Alexandros Gryparis, Rafaella Georgiou, Dionysios Tafiadis

**Affiliations:** 1 Department of Speech & Language Therapy, School of Health Sciences, University of Ioannina, 45 500 Ioannina, Greece; siafaka@uoi.gr (V.S.); alexandros@uoi.gr (A.G.); r.georgiou@uoi.gr (R.G.); tafiadis@uoi.gr (D.T.); 2 Department of Health Sciences, Speech and Language Therapy, European University, 2404 Nicosia, Cyprus; l.voniati@euc.ac.cy

**Keywords:** Distress Thermometer for Parents (DT-P), chronic illnesses, parental distress, pediatric dysphagia, validation, psychometric properties

## Abstract

**Background/Objectives:** The Distress Thermometer for Parents represents an excellent tool for the rapid assessment of emotional distress in parents of children with a variety of chronic diseases. This study aimed to evaluate the efficacy of the Distress Thermometer for Parents in assessing emotional distress in parents and caregivers of children with feeding and swallowing difficulties feeding swallowing disorders or, dysphagia, in the Greek Cypriot community. **Methods:** It involved 200 Greek Cypriot participants: 100 parents/caregivers of children with FSD and 100 parents/caregivers of children without such difficulties. Demographic and clinical data were collected and test–retest reliability was evaluated. **Results:** The DT-P demonstrated excellent reliability (Cronbach’s α = 0.928) and strong test–retest consistency (r = 1.00, *p* < 0.001). ROC analysis showed strong discrimination for detecting distress, with an AUC of 0.76 for parents of children under 24 months and 0.77 for parents of children over 2 years (*p* < 0.01). Parents reported a medium level of distress (M = 3.67, SD = 2.89), with “Emotional Problems” (M = 2.03, SD = 2.28) and “Practical Problems” (M = 1.79, SD = 2.12) contributing most significantly. Independent *t*-tests revealed significantly higher distress levels in parents in the clinical group compared to the parents in the typical group (*p* < 0.001), confirming the DT-P’s sensitivity and validity. **Conclusions:** The Greek version of the DT-P is a reliable and valid tool assessing distress in parents of children with PFD. This study highlights significantly higher distress levels in these parents compared to those of typically developing children, emphasizing the need for targeted support.

## 1. Introduction

Chronic illnesses have become a major focus in global research and healthcare [1,2]. The international literature defines “chronic illnesses or diseases” as health conditions that last for more than three months, including all pathological conditions [3,4]. More specifically, chronic diseases are defined as all chronic conditions that affect the abnormal functioning of the body and may cause disability and death [3,5]. Especially for children, this group include various syndromes, neurodevelopmental conditions, physical disabilities, as well as pathological conditions, which can cause illness and affect a child’s normal abilities and functions [3,6].

The prevalence of pediatric chronic illnesses is increasing, with studies reporting 14–18% of children and adolescents in countries such as the USA and the Netherlands experiencing such conditions [7,8]. These children are also at risk for possible medical complexity, as well as social, educational, and emotional complications underscoring the need for a comprehensive approach to special healthcare, as these illnesses often affect not only the child but also their family structure and functioning [9,10].

Pediatric dysphagia, a feeding and swallowing disorder (FSD), is both a symptom and a consequence of various chronic illnesses, making it a significant and complex challenge in pediatric healthcare [11]. It refers to difficulties in swallowing function among children and is commonly associated with chronic conditions such as cerebral palsy, muscular dystrophy, tumors, encephalopathies, intellectual disabilities, developmental delays, and other neurological disorders [11,12,13]. These conditions impair muscle strength, coordination, or neurological processes, resulting in chronic dysphagia that requires long-term management and multidisciplinary intervention [14,15].

The causative factors may be polymorphic or linked to underlying medical conditions [16], as feeding and swallowing are complex functions regulated by brain activity [17,18]. Pediatric dysphagia may result from disease, surgery, strokes, brain injuries, or trauma, and it is particularly prevalent in premature infants and children with neurological dysfunctions and syndromes [19,20]. In such cases, basic human functions like breathing and feeding, along with communication and social interaction, are often disrupted from an early age [12].

Though not a disease itself, pediatric dysphagia can severely impact a child’s daily life, affecting essential functions and requiring extensive, ongoing care [15,18]. Families must adapt to specialized care routines, which often include the use of feeding tubes and collaboration with speech and occupational therapists [16,19,21]. This level of care reshapes family dynamics and demands resilience [21].

Parenting a child with a neurodevelopmental disorder (NDD) often leads to greater stress compared to raising a typically developing child. Research has consistently shown that parents of children with NDDs experience higher levels of stress [22,23,24,25]. Specifically, studies have highlighted that parents of children with autism spectrum disorder (ASD) report significantly higher levels of stress compared to parents of typically developing (TD) children [26,27]. Additionally, children with ASD tend to exhibit more behavioral and emotional challenges than their typically developing peers, which further contribute to the increased stress levels of their parents [28,29,30,31,32]. Parenting stress occurs when the demands of caregiving exceed a parent’s perceived ability to cope [26,27]. While all parents experience some level of stress, chronic or intense stress can negatively affect family dynamics, parental mental health, and overall well-being. This stress is influenced by multiple factors, including the child’s needs, parental coping strategies, family interactions, and environmental factors, all of which interact and contribute to the overall stress experienced by parents [26,33].

The emotional impact of caregiving for children with chronic diseases has been widely studied, and various tools have been developed to assess parental distress. One such tool is the original Distress Thermometer (DT), which is a valuable instrument for measuring distress levels. The DT uses a rating scale from 0 (indicating no distress) to 10 (indicating extreme distress), allowing individuals to indicate their overall level of distress [34].

Several other tools have been designed to evaluate parental distress in relation to chronic diseases. The Parenting Stress Index (PSI), developed by Abidin (2006), is widely used to measure parental stress, particularly in those caring for children with chronic conditions [35]. Similarly, the Parental Stress Scale (PSS), originally validated by Berry and Jones (1995) and later examined by Whittingham et al. (2013) for parents of children with chronic conditions, provides further insight into stress levels [36,37]. Another tool, the Caregiver Strain Index (CSI), developed by Robinson (1983), assesses the burden experienced by caregivers of children with medical conditions [38]. In addition to these tools, the Pediatric Quality of Life (PedsQL) family impact module, developed by Varni et al. (2001) and later confirmed by Varni et al. (2006), evaluates how childhood illnesses affect family life and parental anxiety [39,40]. Broader measures such as the SF-36, developed by Ware and Sherbourne (1992) and, later on, de Oliveira et al. (2015), assessed the overall impact of caregiving on parental quality of life across the lifespan [41,42]. Furthermore, the Family Stress Scale (FSS), based on the conceptual framework developed by McCubbin and Huang (1989), examines the various stressors experienced by families managing chronic illnesses [43,44].

In light of the aforementioned tools and the broader international research literature, the validation and reliability assessment of parent-reported outcome measures are of critical importance. These instruments not only advance our understanding of parental experiences but also offer standardized approaches to evaluating the psychological and emotional impact of caring for children with chronic health conditions, including pediatric feeding and swallowing disorders. As such, the translation and cross-cultural adaptation of these measures should be regarded as essential to support their clinical relevance and applicability across diverse linguistic and cultural contexts.

Among these established tools, the Distress Thermometer for Parents (DT-P) has emerged as a brief and targeted screening measure designed specifically to assess the distress levels of parents caring for children with chronic illnesses [7,45]. It offers a quick and efficient method for evaluating parental distress, facilitating communication between healthcare professionals and families regarding emotional well-being. The DT-P is widely used to capture the emotional and daily life challenges faced by parents of children with chronic conditions [7,45], enabling timely support and intervention where needed [7].

Recognizing the importance of addressing parental distress, this study aims to assess stress levels among parents of children with chronic diseases, both with and without FSDs, using the DT-P. The Greek version of the DT-P is employed to explore the emotional challenges experienced by parents and caregivers in Cyprus. Through this tool, the study seeks to generate valuable insights that support the development of targeted interventions and improve the well-being of families managing chronic pediatric conditions.

## 2. Methods

### 2.1. Participants

A comprehensive and multifaceted recruitment approach was utilized to engage a broad and diverse sample of parents of children with various pediatric medical conditions (as described in Table 1 and Table 2). A total of 200 parents completed the questionnaire and were categorized into two groups: (a) parents of children with atypical feeding and swallowing abilities and (b) parents of children with typical feeding and swallowing abilities. The study sample was restricted to parents of children aged six months to sixteen years.

Participants were excluded based on the following criteria: absence of a documented feeding and/or swallowing diagnosis, unavailability of parents during the questionnaire re-administration process, completion of the questionnaire by a family member other than the parents, and non-compliance with the specified age range of six months to sixteen years.

### 2.2. Procedure

The initial step in validating and verifying the reliability of the Greek version (DT-P) involved a pilot study with 60 parents/caregivers, including 30 whose children had feeding disorders and 30 whose children did not [46]. These children, aged 3 to 9 years, were attending either mainstream or special schools in Cyprus. All parents completed the DT-P after providing informed consent, and the questionnaire was re-administered 10 days later to assess test–retest reliability. The pilot study was used to evaluate the usability, clarity, and internal consistency of the Greek version of the DT-P; however, no modifications to its structure or scoring were necessary based on the results. The results of the pilot demonstrated statistically significant group differences [t(58) = 3.893, *p* < 0.001], excellent test–retest reliability (r = 1.00, *p* < 0.001), strong internal consistency (Cronbach’s α = 0.830), and good discriminatory ability (AUC = 0.768, *p* < 0.001).

During the second part of the study, 200 parents/caregivers were participants, whose children aged 6 months to 16 years were recruited from private speech therapy clinics or public and special school settings, in Cyprus (as seen in Table 1). The parents/caregivers were responsible for feeding their children and were divided into a clinical group (n = 100) and a control group (n = 100). Principals, school directors, and clinic staff were informed about ethical procedures and parental consent while ensuring the confidentiality and appropriate use of personal information. Parental approval was obtained and data were collected in different settings, including public and special needs schools in Cyprus, university practice intervention clinics, and private speech therapy clinics. The research team conducted interviews with parents/caregivers and reviewed the child’s medical records to collect demographic and clinical data, identify feeding and swallowing issues, and confirm eligibility for participation. Following the adequate collection of the information above, the Distress Thermometer for the parents’ questionnaire was distributed to all participants. Subsequently, the same 200 parents/caregivers were invited to complete the questionnaire again ten days later in order to evaluate the test–retest reliability of the scale.

### 2.3. Instrument

The DT-P questionnaire comprises a thermometer, the “Thermometer Score”, which measures parents’ general level of distress, and a problem list comprising three main components. First, it includes a thermometer scale of 0 to 10, on which parents rate their general anxiety for the last week. A score of 4 or above indicates clinically significant anxiety. The second is a list of problems, which asks parents to identify any problems (for children under two years) or issues (for children aged two years and over) that they have experienced in the last week. These issues are divided into six domains: practical, social, emotional, physical, cognitive, and parenting, with each domain score calculated as the sum of the answers to the questions ‘yes’ (with a score of 1) and ‘no’ (with a score of 0). Finally, the third part includes further questions on the perception of support from others, feelings of misunderstanding, any chronic illness they may have, and whether they wish to speak to a professional about their situation. In the current study, the adapted version of the DT-P for parents of children with chronic disease was used [45]. The problem list consists of 37 items for parents of children under 2 years old (maximum score of 35) and 34 items for parents of children 2 years and older (maximum score of 34). The total score can range from 0 to 45 (for children under 2) or 0 to 44 (for children 2 years and older).

### 2.4. Statistical Analysis

Statistical analysis was performed using IBM SPSS statistical software (version 29.0, Armonk, NY, USA) and Jamovi software (version 29.0). Initially, in this study, we conducted a statistical analysis to assess the distribution of our data [47]. We performed descriptive statistics to summarize key characteristics and employed two tests to evaluate normality: the Kolmogorov–Smirnov test and the Shapiro–Wilk test. The second area of analysis was the DT-P’s psychometric features. A Cronbach alpha of 0.70 was regarded as satisfactory for internal consistency, whereas 0.80 was appraised as good. Furthermore, to determine how strongly two variables are related and whether that relationship is positive or negative, the thermometer and DT-P issue domain scores were correlated using the Pearson r correlation method. Values ranged from −1 (a perfect negative link) to 1 (a perfect positive link). The Kaiser–Meyer–Olkin (KMO) test and Bartlett’s test of sphericity were used to calculate the factorability of our data. Thirdly, an examination of receiver operating characteristics (ROC) was conducted. ROC analysis is a commonly utilized method for making clinical decisions, especially ones utilizing diagnostic questionnaires. It generates a graph that can be used to determine patient classification cut-off points. The optimal cut-off was computed using the Yuden Index. The discriminatory potential of the DT-P as a tool for tracking elevated levels of distress, including the establishment of a cutoff score, was examined using an ROC analysis. The DT-P’s discriminatory power can be defined as strong, moderate, or low, determined by whether the area under the ROC curve is greater than 0.75, less than 0.5, or 0.5–0.75, respectively. For every score (0–10) on the thermometer, positive and negative predictive values, specificity, and sensitivity were computed.

## 3. Results

### 3.1. Descriptive Statistics

This study included 200 Greek Cypriot parents and caregivers, 162 of whom were mothers. The clinical group had parents of 72 boys and 28 girls, while the typically developing group consisted of parents of 44 girls and 56 boys (Table 1).

The children’s median age in the clinical DT-P group was 7.60 (IQR: 4.2–10.9) and 6.85 (IQR: 4.25–10.925) in the DT-P control group.

The feeding and swallowing difficulties observed in children within the clinical group were attributed to various underlying conditions, including acquired disorders, developmental disorders, genetic syndromes, cerebral palsy, and other medical conditions (detailed in Table 2).

### 3.2. Psychometric Characteristics of the DT-P

#### 3.2.1. Internal Consistency and Validity

Table 3 provides a detailed overview of the descriptive statistics and psychometric properties of the Distress Thermometer for Parents (DT-P), across each domain, including 200 parent–caregiver participants, of Greek Cypriot children experiencing FSDs.

The outcomes detected a medium level of distress, on average, and a mean score of 3.67 with a standard deviation of 2.89. The problem checklist has six domains, and parents–caregivers were asked to respond with either “yes” or “no”. The internal consistency results of each domain are comparable with the Cronbach α values seen in other DT-P studies. Thus, the “Practical Problems” domain identified challenges encountered during daily living situations. It was evaluated with an average of 1.79 and a standard deviation of 2.12. The seven elements in this domain had Cronbach’s alpha of 0.827, indicating strong internal consistency (as seen in Table 3).

The “Family/Social Problems” domain covered difficulties in family and social interactions. The mean score in this domain was 0.49, with a standard deviation of 0.92, thus representing less anguish in this domain. The internal consistency of Cronbach’s alpha = 0.676 and was close to the ideal but below the cut-off point, yet still acceptable. This domain had four items with scores that ranged from 0 to 4. The “Emotional Problems” domain calculated emotional difficulties; the mean was 2.03, and the SD was 2.28. Internal consistency was high, with a Cronbach’s alpha of 0.820. There were nine items, and the scores ranged from 0 to 9.

The domain relating to “Physical Problems” was the measurement of actual physical problems experienced by the parents, which had a score of 1.84 ± 2.05, and good internal consistency with a Cronbach’s alpha of 0.825; it had seven items whose scores ranged between 0 and 7. The “Problems with Cognition” domain rated the cognitive problems and expressed a mean of 0.54 with a standard deviation of 0.77. The internal consistency was lower at a Cronbach’s alpha of 0.674, but it was still considered acceptable. The two items in this domain scored between 0 and 2 (Table 3).

Also, “Parenting (Children ≥ 2 years old)” assessed issues related to parenting children who were 2 years old or older, noting a mean score of 0.97 and an SD of 1.46. With five items and a Cronbach’s alpha of 0.805, the internal consistency was good. The items’ scores ranged from 0 to 5. Finally, “Parenting (Children < 2 years old)” had a mean of 1.67, with a standard deviation of 2.15, concerning problems of parenting in children less than 2 years old. The internal consistency for this domain was excellent, with a Cronbach’s alpha of 0.880 and six items, with scores ranging from 0 to 6 (Table 3).

The overall score for all domains demonstrated excellent internal consistency, with a Cronbach’s alpha of 0.928 across all participants. This result indicated that the DT-P, in its Greek version, when given to parents and caregivers of children experiencing feeding and swallowing, is a very reliable instrument for measuring the degree of distress of parents who have children with feeding or swallowing problems in the Greek Cypriot population. The results indicated that parents do suffer distress at different levels in various domains and that the major contribution to the overall level of distress comes from the domains of emotional and practical problems (Table 3).

In summary, the total score of all the domains had an excellent internal consistency (Cronbach α ≥ 0.928). Also, the total score (with parenting children younger than 2) domain appeared to have excellent internal consistency (Cronbach α ≥ 0.934), and the total score (with parenting with children 2 and older) had (Cronbach α ≥ 0.936) as shown in Table 3.

#### 3.2.2. Sensitivity and Specificity

To evaluate the DT-P’s efficacy in identifying elevated levels of distress, we used the receiver operating characteristic (ROC) analysis. This method is widely used in healthcare decision-making, including the validation of screening questionnaires, due to its ability to measure a tool’s diagnostic accuracy.

**Figure 1:**
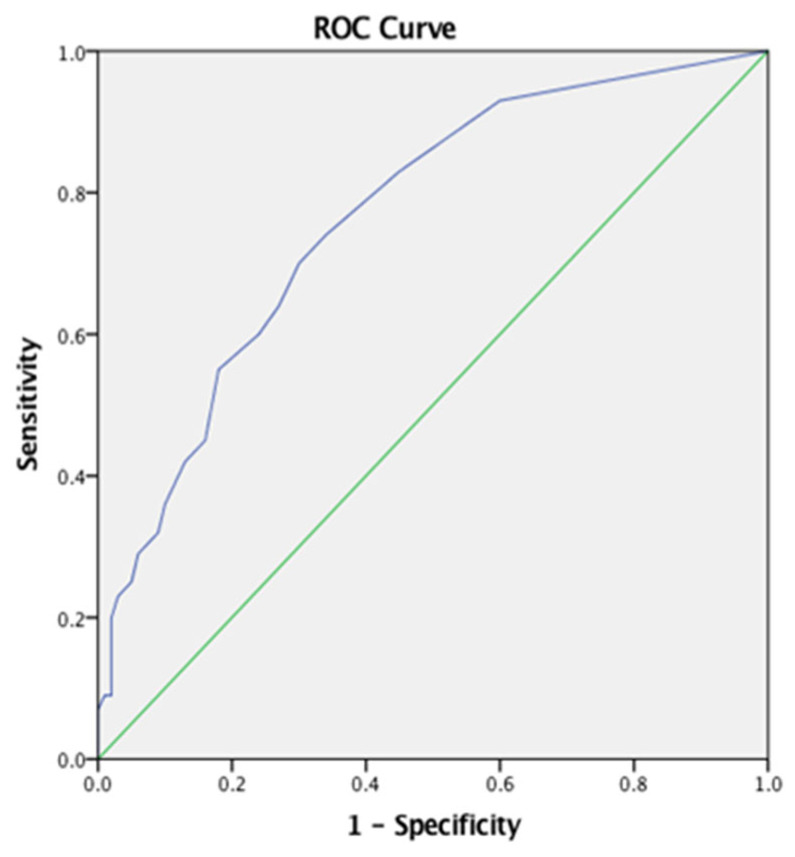
ROC curve for total DT-P score between parents/caregivers with and without feeding and/or swallowing disorders.

The ROC analyses showed that the DT-P total score had a strong discrimination capacity with an area under the curve (AUC) of 0.765 [SE = 0.033, 95% (0.70, 0.83), *p* < 0.01], with the optimal cut-off point equal to four <4> (sensitivity = 0.70 and 1-specificity = 0.30) between the parent/caregiver groups with and without feeding and/or swallowing disorders, as shown in Figure 1.

#### 3.2.3. Comparison of Means Between Groups

An independent-sample *t*-test examined the comparison of the distress levels (measured by DT-P) between the clinical and the typical groups across the various domains. The results obtained in all domains, except “Parenting < 2 years old child” and “Total score < 2 years old child,” showed significant differences. The clinical group indicated higher incidents of practical, emotional, family/social, physical, and cognitive problems than the typical group. For instance, the mean score regarding practical problems in the clinical group was 2.74, while that for the typical group of children was 0.83, demonstrating that the former experiences more practical problems than the latter. These differences, according to the t-values and *p*-values, are statistically significant, with *p*-values less than 0.001, in all cases; hence, the two groups are statistically very different. Additionally, most of the effect sizes (eta) represented were at least moderate to large. In particular, the overall distress thermometer score had an eta of 0.272, indicating the big difference in overall distress between these two groups (Table 4).

#### 3.2.4. Construct Validity

All values of the Kaiser–Meyer–Olkin (KMO) Measure of Sampling Adequacy exceeded 0.5, thus indicating that the sample size was enough to perform factor analysis. Specifically, some domains, like practical problems, had 0.837, while physical problems had a value of 0.849, indicating excellent sampling adequacy. Bartlett’s test of sphericity for all factors is highly significant (*p* < 0.001); hence, the variables are correlated enough to proceed with factor analysis. The percentage of variance accounted for by the factors ranges from 32.372 to 75.551. Cognitive problems account for the highest percentage of variance at 75.551%. This result indicated that these identified factors account for significant variance in the distress scores (Table 5).

## 4. Discussion

The Greek version of the DT-P was validated and culturally adapted for use with the Greek Cypriot population to measure the distress levels in parents and caregivers of children with FSDs. The findings were consistent with previous research on the DT-P’s psychometric properties and emphasized cultural differences relevant to the Greek Cypriot context. The psychometric qualities of the Greek version of the DT-P were constructed and assessed using data gathered from 200 Greek Cypriot parents/caregivers of children with or without FSDs. Its cultural adaptability and clinical implications are discussed in the following sections.

The Greek DT-P demonstrated excellent psychometric properties, comparable to its Dutch and its original version [7,45]. The internal consistency of the Greek DT-P was high across most domains, with Practical Problems (α > 0.80) and Emotional Problems (α > 0.85) showing strong reliability. This is consistent with prior studies showing how parents of children with chronic illnesses manage emotional strain and day-to-day difficulties [7]. Though the Family/Social Problems and Cognitive Problems domains had slightly lower Cronbach’s alpha values (0.676 and 0.674), these remained within acceptable limits and aligned with previous validation findings [45]. The total score’s internal consistency (α ≥ 0.928) further supports the tool’s reliability.

Construct validity was supported through the CFA and KMO measures of sampling adequacy. KMO values exceeded 0.8 in most domains, and Bartlett’s test of sphericity showed significant inter-variable correlations, validating the tool’s factorial structure. These results confirm that the DT-P captures the multifaceted nature of parental/caregiver distress, especially practical, emotional, and physical burdens [7,45].

Compared to the Dutch and original versions of the DT-P, this validation study maintained robust psychometric properties reflecting cultural distinctions. For example, the domain Family/Social Problems displays slightly lower distress levels, which may indicate stronger familial support systems in Greek Cypriots, an interpretation that needs further exploration. Emotional and Practical challenges were the most prominent sources of distress, echoing findings in other studies [7,45]. These domains are suggestive of the day-to-day struggles faced by parents of children with chronic conditions, including FSDs.

Notably, parents of children with FSDs experienced higher distress levels compared to parents of typically developing children (mean DT-P scores of 5.17 for the clinical group and 2.16 for the control group). This confirmed the emotional and practical impact of caring for a child with an FSD, aligning with the existing literature on caregiver burden in pediatric dysphagia [7,45].

Significant differences were observed in the levels of distress between the clinical and control groups across all domains, except for parenting children under 2 years, highlighting the increased challenges faced by parents of children with FSDs.

The largest domain differences were in Practical Problems (mean score: 2.74 vs. 0.83) and Emotional Problems (mean score: 2.88 vs. 1.18), which had the largest differences, reflecting the specific burdens associated with managing these children’s complex needs, not only because of dysphagia but also because of the underlying neurodevelopmental or other disorders or syndromes. These findings are consistent with the results from Haverman et al. (2013) and van Oers et al. (2017), reinforcing the DT-P as a useful tool in differentiating between parents/caregivers experiencing varying levels of distress [7,45].

Kyranou et al. (2020) validated the Greek version of the Distress Thermometer (DT) in patients with cancer, comparing it to clinical interviews for depression [48]. Their study reported an AUC of 0.79, recommending a cut-off score of 4 to prioritize sensitivity [48]. This aligns with the present study, which also supports using a cut-off of 4 to identify at-risk individuals, thereby improving sensitivity. These findings collectively support the DT-P questionnaire’s strong discriminatory power and reliability across various populations and contexts.

Additionally, ROC analysis demonstrated strong discriminatory power in identifying parents/caregivers experiencing elevated levels of distress due to FSDs. These results are consistent with findings from both the original and Dutch versions of the DT-P, underscoring the tool’s diagnostic validity and reinforcing its applicability across diverse cultural contexts [7,45].

In general, the results in Table 4 and Table 5 confirmed that the DT-P is a reliable and valid tool for measuring difference in levels of distress between parents and caregivers of children with and without FSDs. The results of CFA support the factorial structure and sampling adequacy of the DT-P, thus confirming its usefulness in measuring parental–caregiver distress [7,45].

By recognizing pediatric dysphagia as part of the spectrum of chronic illnesses, healthcare providers and policymakers can better advocate for resources that enhance the quality of life for affected families. To further address the growing prevalence of pediatric dysphagia and related chronic illnesses, healthcare systems must adopt integrated approaches. Thus, early diagnosis, multidisciplinary treatment teams, and family-centered care models can prioritize developmental and emotional well-being.

### 4.1. Implications

The Greek DT-P is shown to be of clinical and research value in the Cypriot population, particularly for indicating distress among parents/caregivers of children with FSDs. The DT-P can identify specific areas of difficulty, enabling the implementation of targeted interventions, such as emotional support programs or practical assistance, to address challenges associated with feeding practices [7]. Therefore, this can be of immense use in clinical and research aspects and for policymakers aiming to address parental/caregiver well-being in pediatric care. Applying such assessments can inform policymakers in developing comprehensive support systems, including mental health services and caregiver support initiatives [49,50]. This aligns with the growing emphasis on comprehensive pediatric care that addresses child and parent/caregiver well-being [51,52]. This confirms the Greek DT-P as an effective tool for identifying parental distress, bridging research and clinical application while respecting cultural sensitivities.

### 4.2. Strengths

This is the first research study using the DT-P in Greek Cypriot parents/caregivers of pediatric patients who experience FSDs. It was established that there were significant differences in the level of distress between the two groups. This difference established that the DT-P has the strength of capturing the unique difficulties experienced by parents/caregivers in the clinical group, thus validating its practical usefulness in distinguishing between different levels of distress.

Moreover, the cultural adaptation of the DT-P and its validation in the Cypriot context ensured that it is culturally relevant. As such, this tool becomes especially useful in facilitating appropriate communication and more effective support by health professionals and clinicians working with Greek-speaking parents/caregivers.

### 4.3. Limitations

Despite this study’s many strengths, certain drawbacks should be noted. First, while the sample size of 200 Cypriot parents/caregivers is a strength, it may not fully represent the broader Greek Cypriot pediatric population. This raises the possibility of selection bias; hence, a bigger sample size with increased representativity is needed if generalization to other samples is to be attempted. Cultural bias may also be present, as parental perceptions of distress could be influenced by culturally specific attitudes toward caregiving and child health.

This study was primarily based on self-report data from parents, perhaps reflecting response biases in terms of socially desirable responses, or even a recall of the existence of FSDs, in children. Additionally, even if the DT-P showed strong psychometric properties, the lack of a gold standard against which to compare it to was considered a limit when assessing parents’/caregivers’ distress. Criterion validity may be further pursued by comparing the DT-P with other established tools for measuring parental distress in the same populations.

Furthermore, the gender distribution of respondents was imbalanced, with a greater proportion of mothers participating in this study. This likely reflects the Cypriot context, because mothers are more often the primary caregivers and more engaged in health-related activities and research participation. However, this imbalance may limit the generalizability of the findings to all caregivers, particularly fathers or other guardians, whose experiences of distress may differ. Future studies should aim for a more balanced gender representation to ensure a fuller understanding of caregiver perspectives and reduce potential gender-related response bias.

Finally, the absence of longitudinal follow-up limits understanding of how caregiver distress may evolve over time. Regardless of these limitations, the present study has indicated that the DT-P can be reliably used in the Greek clinical setting, and it has identified the optimal cut-off score for the Greek Cypriot population.

### 4.4. Future Directions

Future research should extend to larger samples, including participants from different Greek-speaking regions, to further augment generalizability. Conducting such studies will broaden representation and would also help address potential cultural bias and improve the external validity of the DT-P. If further validation is carried out, there would be stronger evidence for the tool’s reliability and validity in diverse contexts. Comparative studies could additionally help identify the DT-P’s relativity among other available tools while recognizing its explicit benefits. Longitudinal studies may identify the sensitivity of the DT-P to changes in the level of distress over time. Such studies could also provide further insight into the instrument’s usefulness in monitoring progress and assessing the effectiveness of intervention strategies while reducing parental distress.

In addition, future research should be focused on implementing the DT-P into routine clinical practice, where it could facilitate more effective communication between healthcare professionals and parents/caregivers. Understanding its utility in supporting timely and targeted interventions could enhance its practical impact on parental/caregiver well-being. Additionally, a digital version of the DT-P would be a worthwhile area of exploration. Such adaptations could increase accessibility and user-friendliness, particularly for caregivers with limited time or resources to complete paper-based questionnaires. A digital format would support wider implementation and streamline data collection in both clinical and research environments.

## 5. Conclusions

The Greek version of the DT-P has been demonstrated to be a valid and reliable instrument for assessing distress among parents and caregivers of children with feeding and swallowing difficulties in Cyprus. This tool effectively differentiates between clinical and typical populations, underscoring its significance in clinical practice. Its ease of application and cultural relevance make it an invaluable resource for health professionals in evaluating and managing parental or caregiver distress.

While the DT-P serves as a useful tool to identify areas contributing to parental stress, it is essential to note that it does not replace a comprehensive clinical evaluation by health professionals. Research indicates that the DT-P can address various significant issues that distress parents and caregivers of children facing feeding and swallowing disorders (FSDs). The level of distress experienced by parents and caregivers can adversely affect their decision-making capabilities and adherence to treatment plans. Therefore, utilizing the DT-P should complement broader treatment strategies.

The findings affirm the Greek version of the DT-P as a robust tool for assessing parental distress within the Greek Cypriot community. With its strong psychometric properties, cultural adaptability, and clinical relevance, the DT-P holds substantial promise for enhancing support services for parents and caregivers. By effectively identifying those experiencing distress, this instrument can bridge the gap between research and clinical application while honoring cultural sensitivities.

The Greek version of the DT-P is a valid, reliable measure of distress among parents/caregivers of children with feeding and swallowing difficulties in Cyprus. It distinguishes between clinical and typical groups, showing its relevance for use in clinical practice. It is easy to apply and culturally relevant; therefore, this instrument will be very useful in health professionals’ assessment and management of parental/caregiver distress. It is a robust instrument to measure for future application and advancement to support families whose children struggle with swallowing and feeding.

One of the most important aspects of screening tools such as the DT-P is that they are straightforward to complete. While screening can help identify the areas that most hinder parental/caregiver stress, it is not a diagnostic process and cannot substitute a health professional’s clinical evaluation. As this study and numerous others have demonstrated, it may also tackle an array of significant problems distressing parents/caregivers of children experiencing FSDs. Parents’/caregivers’ ability to make decisions and adhere to treatment plans are impacted by distress, and this process must emerge in addition to further aspects of treatment. With its clinical relevance, cultural adaptability, and strong psychometric qualities, the DT-P has immense potential to enhance parent/caregiver support services even more. Thus, the well-being of the children can be improved by identifying parents/caregivers who are experiencing distress, bridging research and clinical application while respecting cultural sensitivities. It provides an expansible model for adapting psychosocial instruments across diverse populations.

## Figures and Tables

**Table 1:** Demographic characteristics of the sample.

	Clinical Group (n = 100)	Control Group (n = 100)	*p* Value
	Median (IQR)	Median (IQR)	
Children’s Age (years)	7.60 (4.25–10.90)	6.85 (4.25–9.40)	0.407 +
Parents’ Age (years)			
Maternal Age	39.00 (35.00–42.00)	37.00 (35.00–41.50)	0.498 +
Paternal Age	41.00 (36.00–46.00)	40.00 (37.00–43.00)	0.980 +
Parents’ Gender, N (%)			
Male	25 (25.0%)	13 (13.0%)	0.498 ++
Female	75 (75.0%)	87 (87.0%)	0.980 ++
Children’s Gender, N (%)			
Male	72 (72.0%)	56 (56.0%)	
Female	28 (28.0%)	44 (44.0%)	

Abbreviations: IQR: interquartile range; + independent-sample *t*-test; ++ chi-square; *p*-value < 0.05.

**Table 2:** Medical diagnosis and feeding/swallowing disorders of the children in the clinical group.

Medical Group N (%)	Diagnosis
Acquired Disorders N = 8 (8%)	Cerebellar Atrophy (N = 5), Left Side Lobotomy (N = 1), Medial Agenesis (N = 1), PKU (N = 1),
Developmental Disorders N = 44 (44%)	ASD (N = 40), SLI (N = 2), Neurodevelopmental Disorder (N = 2),
Genetic Syndromes N = 15 (15%)	Fragile X Syndrome (N = 2), Coffin-Siris Syndrome (N = 1) DiGeorge Syndrome(N = 1), Down Syndrome (N = 5), Dandy-Walker Syndrome (N = 1), Mowat Wilson Syndrome (N = 1), Rett Syndrome (N = 2), 1q44 Syndrome (N = 1), ArCapa Syndrome (N = 1)
Cerebral Palsy Ν = 15 (15%)	Cerebral Palsy (N = 15)
Other Medical Conditions N = 18 (18%)	Hard of Hearing (N = 1), Severe delays in Development (N = 17)
Feeding/Swallowing Disorder, N (100%)	Oral Sensory Feeding Disorder 41 (41%) Oral Motor Feeding Disorder 43 (43%) Oropharyngeal Dysphagia 16 (16%)

Abbreviations: ASD: autism spectrum disorder, PKU: phenylketonuria, SLI: Specific Language Impairment.

**Table 3:** Descriptive statistics of DT-P for all participants (n = 200).

DT-P	n	M	S.D.	Cronbach’s α	No. of Items	Possible Scores		Observed Scores	
						Min	Max	Min	Max
Thermometer score (overall distress)	200	3.67	2.89			0	10	0	10
Practical problems	200	1.79	2.12	0.827	7	0	7	0	7
Family/social problems	200	0.49	0.92	0.676	4	0	4	0	4
Emotional problems	200	2.03	2.28	0.820	9	0	9	0	8
Physical problems	200	1.84	2.05	0.825	7	0	7	0	7
Cognitive problems	200	0.54	0.77	0.674	2	0	2	0	2
Parenting ≥ 2 years old child	188	0.97	1.46	0.805	5	0	5	0	5
Parenting < 2 years old child	12	1.67	2.15	0.880	6	0	6	0	6
Total score (5 domains)	200	6.68	6.78	0.928	29	0	29	0	27
Total score ≥ 2 y (6 domains)	188	7.70	7.70	0.934	34	0	34	0	31
Total score < 2 y (6 domains)	12	7.50	7.82	0.936	35	0	35	0	25
Enough support (yes)	200	192	96.0%						
Wish for referral (yes/maybe)	200	57	28.5%						

Abbreviations: M: mean; S.D.: standard deviation; Min: minimum; Max: maximum.

**Table 4:** Independent-sample *t*-test for DT-P between clinical and typical groups.

	Clinical			Typical			t	df	*p*-Value	Eta
	n	Mean	S.D.	n	Mean	S.D.				
Practical problems	100	2.74	2.28	100	0.83	1.43	7.11	166.50	<0.001	0.203
Emotional problems	100	2.88	2.46	100	1.18	1.71	5.67	176.33	<0.001	0.140
Family/social problems	100	0.82	1.13	100	0.15	0.46	5.49	130.57	<0.001	0.132
Physical problems	100	2.42	2.14	100	1.26	1.77	4.17	191.31	<0.001	0.081
Cognitive problems	100	0.77	0.85	100	0.31	0.60	4.42	177.58	<0.001	0.090
Parenting ≥ 2 years old child	93	1.55	1.61	95	0.40	1.04	5.82	156.77	<0.001	0.155
Parenting < 2 years old child	7	2.57	2.37	5	1.40	0.40	2.21	8.15	0.057	0.271
Total score (5 domains)	100	9.63	7.12	100	3.73	4.90	6.83	175.48	<0.001	0.190
Total score ≥ 2 y (6 domains)	93	11.33	8.13	95	4.15	5.23	7.19	156.50	<0.001	0.219
Total score < 2 y (6 domains)	7	10.14	8.73	5	3.80	4.97	1.45	10.00	0.177	0.174
Thermometer score (overall distress)	100	5.17	2.53	100	2.16	2.42	8.61	198.00	<0.001	0.272

**Abbreviations**: M, mean; S.D., standard deviation; t, independent-sample *t*-test; Eta, eta coefficient; *p* < 0.005.

**Table 5:** Confirmatory Factor Analysis (CFA), KMO Measure of Sampling Adequacy, and Bartlett’s test of sphericity for all factors used in this study.

Factor	KMO Measure of Sampling Adequacy	Bartlett’s Test of Sphericity			Percentage of Variance Explained (%)
		Approx. Chi-Square	df	*p*-Value	
Practical problems	0.837	426.071	21	<0.001	49.162
Family/social problems	0.653	140.833	6	<0.001	51.343
Emotional problems	0.828	527.417	36	<0.001	41.781
Physical problems	0.849	433.768	21	<0.001	49.655
Cognitive problems	0.500	59.774	1	<0.001	75.551
Parenting ≥ 2 years old child	0.811	283.110	10	<0.001	56.373
Parenting < 2 years old child	---	---	---	---	67.696
Total score (5 domains)	0.872	2728.621	406	<0.001	34.265
Total score ≥ 2 y (6 domains)	0.865	3192.034	561	<0.001	32.372
Total score < 2 y (6 domains)	---	---	---	---	---

**Footnote**: KMO stands for Kaiser–Meyer–Olkin Measure of Sampling Adequacy.

## Data Availability

Due to ethical restrictions, the data generated during or analyzed in the current study are not publicly available. For consideration, all data queries and requests should be submitted to the corresponding author, Andri Papaleontiou.
